# Nucleic Acid Prevalence of Zoonotic *Babesia* in Humans, Animals and Questing Ticks, a Systematic Review and Meta-Analysis

**DOI:** 10.3390/tropicalmed8030132

**Published:** 2023-02-22

**Authors:** Xiao-Yan Yao, Shao-Qi Yu, Na Tian, Fei Wang, Shi-Zhu Li, Lan-Hua Li

**Affiliations:** 1 School of Public Health, Weifang Medical University, Weifang 261053, China; 2 Weifang City Center for Disease Control and Prevention, Weifang 261061, China; 3 National Institute of Parasitic Diseases, Chinese Center for Disease Control and Prevention, Shanghai 200025, China; 4 Key Laboratory of Parasite and Vector Biology, Ministry of Health, WHO Collaborating Center for Malaria, Schistosomiasis and Filariasis, Shanghai 200025, China

**Keywords:** *Babesia*, zoonotic, tick-borne disease, prevalence, nucleic acid testing, systematic review

## Abstract

**Background:** Zoonotic *Babesia* infections are an emerging public health threat globally. The geographical distribution, animal reservoirs and tick vectors vary greatly across *Babesia* species, and estimations of prevalence reported in works within the literature are also quite different. Better prevalence estimates and identification of moderators are needed to understand the global transmission risk of different zoonotic *Babesia* species, and to provide crucial background information for the diagnosis, treatment and control of zoonotic babesiosis. **Methods:** We conducted a systematic review and meta-analysis to determine the global nucleic acid prevalence of different zoonotic *Babesia* species in humans, animals and ticks. Relevant publications were obtained from several electronic databases and grey literature up to December 2021. Articles were included if they were published in English or Chinese and reported the nucleic acid prevalence of zoonotic *Babesia* species in humans, animals or ticks. The pooled estimates of prevalence were determined using a random effect model. Heterogeneity was investigated using subgroup analyses and random effect meta-regression models. **Results:** Of 3205 unique studies, 28 were included by the systematic review of zoonotic *Babesia* for humans, 79 for animals and 104 for ticks. The results showed overall pooled estimates of nucleic acid prevalence for the following: *B. microti*—1.93% (0.32–4.69%) in humans; *B. microti*—7.80% (5.25–10.77%), *B. divergens*—2.12% (0.73–4.08%) and *B. venatorum*—1.42% (0.30–3.16%) in animals; and *B. microti*—2.30% (1.59–3.13%), *B. divergens*—0.16% (0.05–0.32%), and *B. venatorum*—0.39% (0.26–0.54%) in questing ticks. The type of population, animal reservoir or tick vector, detecting method and continent were moderators possibly associated with heterogeneity, yet the remaining heterogeneity that was not explained was still substantial (all QE *p* values < 0.05). **Conclusions:** *B. microti* is the most prevalent and widely distributed zoonotic *Babesia* species globally. The wide range of suitable animal reservoirs and potential transmission vectors and high prevalence in animals and ticks may contribute to the worldwide distribution of *B. microti*. Other zoonotic *Babesia* species were relatively less prevalent and were reported in quite limited areas.

## 1. Introduction

Babesiosis is one of the most common tick-borne diseases in wild animals and livestock, caused by intraerythrocytic protozoa of the genus *Babesia*. *Babesia* species are distributed worldwide and considered to be the second most commonly found parasites in the blood of mammals after trypanosomes [1,2]. More than 100 species of *Babesia* have been found all over the world, most of which can only infect animals. However, several *Babesia* spp. can also infect humans and cause human babesiosis, among which *B. microti*, *B. divergens* and *B. venatorum* (previously referred to as *Babesia* sp. EU1) are the most common zoonotic species [2,3]. Other potential zoonotic species include *B. duncani*, *B. crassa*, *Babesia* sp. TW1, *Babesia* sp. XXB/Hangzhou (China) and *Babesia* sp. KO1 (Korea) [4,5].

Understanding the infection rate of zoonotic *Babesia* in humans, animals and ticks can provide crucial background information for the diagnosis, treatment and control of human babesiosis. However, prevalence estimates in the literature vary greatly across different studies [6]. Reported prevalence estimates of *Babesia* might differ because of heterogeneity among different detecting methods [7], or subgroups of people (e.g., people with or without special risk of tick bites) [8] or animal or tick species. Prevalence variation in animals might also result from the type of sample (e.g., blood or tissue) [9]. Furthermore, whether the instar of ticks influences the detection of *Babesia* in animals or ticks is unclear. Recently, several studies have systematically reviewed the prevalence of zoonotic *Babesia* species in humans, animals and ticks [10,11,12,13]. However, the prevalence of different zoonotic *Babesia* species, diverse detecting methods (nucleic acid testing or serological testing) for human or animal infection, and different infesting status of ticks (questing or infesting) were synthesized together in previous systematic reviews. Geographical distribution, animal reservoirs and tick vectors vary greatly across *Babesia* species. Serological testing is limited in differentiating active from convalescent infections and in avoiding cross-reactivity between species [14], yet a nucleic acid-positive result reflects active infection and makes more sense regarding pathogen transmission [15]. Moreover, positive rates of infesting ticks cannot reflect the transmission capability because it might be related to the infection status of animal reservoirs [16]. Therefore, in this study, we aimed to determine the best estimates of nucleic acid prevalence of different zoonotic *Babesia* species in humans, animals and questing ticks, and to identify the extent to which the aforementioned moderators account for heterogeneity among studies.

## 2. Methods

### 2.1. Search Strategy and Selection Criteria

A systematic literature search was carried out to identify all studies reporting infections of the known zoonotic *Babesia* species in humans, ticks and animals from inception to December 31, 2021, in the following electronic databases: PubMed, Embase, Web of Science, Scopus, Elton B Stephens Company (EBSCO), Chinese National Knowledge Infrastructure database (CNKI), SinoMed and Chinese Wanfang database (CWFD). The following terms in English and Chinese were used in the full-text search: (“*Babesia microti*” or “*Babesia divergens*” or “*Babesia venatorum*” or “*Babesia* sp. EU1” or “*Babesia duncani*” or “*Babesia* sp. WA1” or “*Babesia crassia*” or “*Babesia* sp. XXB/HangZhou” or “*Babesia* sp. KO1” or “*Babesia* sp. TW1”). Moreover, the reference lists of studies deemed to be relevant were also manually checked for additional relevant research not indexed by these databases.

After removing duplicates, two independent reviewers (Yao XY and Yu SQ) screened all titles and abstracts identified from the database search, with support from a third reviewer (Tian N). Then, full-text articles were assessed for inclusion by the same reviewers. All studies included were published in English or Chinese, and were observational epidemiological studies reporting the nucleic acid infection rate of known zoonotic *Babesia* species in humans, animals or ticks. Studies were excluded if they were non-primary research articles, letters to the editor, case reports or case series, non-epidemiological studies, not reporting nucleic acid prevalence, or with sample size fewer than 20 [17]. According to Gad Baneth and colleagues, *B. vulpes* sp. nov. should replace the use of synonyms such as B. microti-like, Babesia cf. microti, B. annae and Babesia Spanish dog isolate. [18,19]. Therefore, studies were excluded if they reported the prevalence of *Babesia* cf. *microti* or *B. microti*-like instead of *B. microti*. For prevalence of *Babesia* in ticks, infections of infesting ticks were excluded because they might be related to the infection status of animal reservoirs.

### 2.2. Data Extraction and Quality Assessment

Two authors (Yao XY and Yu SQ) separately assessed the risk of bias of all included studies of human *Babesia* infections using the Hoy Risk of Bias Tool [20,21]. This tool provides a summary score representing the risk of bias on the basis of ten items, each given a score of 0 or 1 for the absence or presence of bias. A summary score of 0–3 indicated a low risk of bias, 4–6 a moderate risk of bias, and 7–10 a high risk of bias. Disagreements were reconciled by the third reviewer (Tian N). Risk of bias assessments for studies of *Babesia* infection in animals or ticks were not performed due to lack of an assessment tool.

One reviewer (Yao XY) extracted data using a standardized data collection form. The extracted data were then verified by a second reviewer (Yu SQ) for accuracy. Information about the following variables was extracted: *Babesia* species, title, first author, publication year, study design, language, research location (country and continent), exact detecting method (PCR, nested PCR or real-time PCR), sample size, number of positives, population type (high-risk population or general population) for infections in humans, type of animal (the taxonomic order of animal host) and sample (blood or tissue) for animal infections, and species, instar and infesting status of ticks for infections in ticks (Tables S1–S3).

For population type, high-risk population refers to people with a high or known possibility of being exposed to ticks, such as people with a history of tick bites (including people infected with other tick-borne pathogens), foresters, livestock keepers, veterinary practitioners or hunters; while the general population includes local residents, blood donors or other people without excess known risk of being exposed to tick bites.

### 2.3. Statistical Analysis

Before pooled estimates were calculated, the double arcsine transformation was used to correct for non-normally distributed raw proportions outside of the range 0.2 to 0.8 [22]. Statistical heterogeneity across studies was estimated using the I^2^ statistic, and its significance was determined using Cochran’s Q test’s *p* value [23]. I^2^ is defined as the ratio of true heterogeneity to total observed variation [24]. If the heterogeneity is statistically significant, a random effect model is used for meta-analysis; otherwise, a Mantel–Haenszel fixed-effect model is adopted [25]. I^2^ value values of 25%, 50% and 75% represent low, medium and high heterogeneity, respectively.

Following the results of the heterogeneity test, a meta-analysis with a random effect model was used in this study to estimate the pooled nucleic acid prevalence of each zoonotic *Babesia* species in humans, animals and ticks. Subgroup and meta-regression analyses were used to explore the potential source of heterogeneity across studies and assess the effects of moderators. For infections in humans and animals, three moderators were considered in subgroups and meta-regression analyses, including continent of the study performed, exact nucleic acid testing method and type of population or animal. For infections in ticks, exact nucleic acid testing method, tick species and instar of ticks were considered. To ensure adequate statistical power for subgroup analyses and meta-regression, meta-analyses were merely conducted for conditions with at least ten data points [26].

I^2^, R^2^, QM and QE statistics were used to quantify heterogeneity and explain the results of moderator analyses [17]. R^2^ is the proportion of true heterogeneity that can be explained by the moderator; the QM statistic and its *p* value show the significance of the moderator in explaining heterogeneity; and the QE statistic and its *p* value show whether the residual heterogeneity (heterogeneity that is unaccounted for by the moderator) is statistically significant.

The presence of potential publication bias was estimated using funnel plots. Funnel plot asymmetry was further assessed by Egger’s test [27,28]. To test the robustness of pooled prevalence estimates, several sensitivity analyses were performed in our study. First, we conducted outlier analyses to determine the influence of outliers on the pooled estimates. Baujat plots and studentized residual inspections were used to detect outlier studies. Studies appearing in the top right quadrant of the Baujat plot or with studentized residuals larger than 3 in absolute value were considered to be outliers [29,30]. After removing identified outliers, the overall pooled prevalence estimates were re-calculated and compared with the main findings. We also ordered studies by precision in forest plots to visually illustrate individual study effects on the pooled estimates. Finally, we examined whether excluding smaller-sample data points (i.e., lowest quintile of each *Babesia* species infection) showed findings similar to the main results.

All statistical analyses were performed using the meta, metafor and weightr packages in R software. For all tests, *p* values of less than 0.05 were considered to be statistically significant. This systematic review is registered with PROSPERO, identifier CRD42022352024.

## 3. Results

### 3.1. Literature Search and Selection

**Figure 1:**
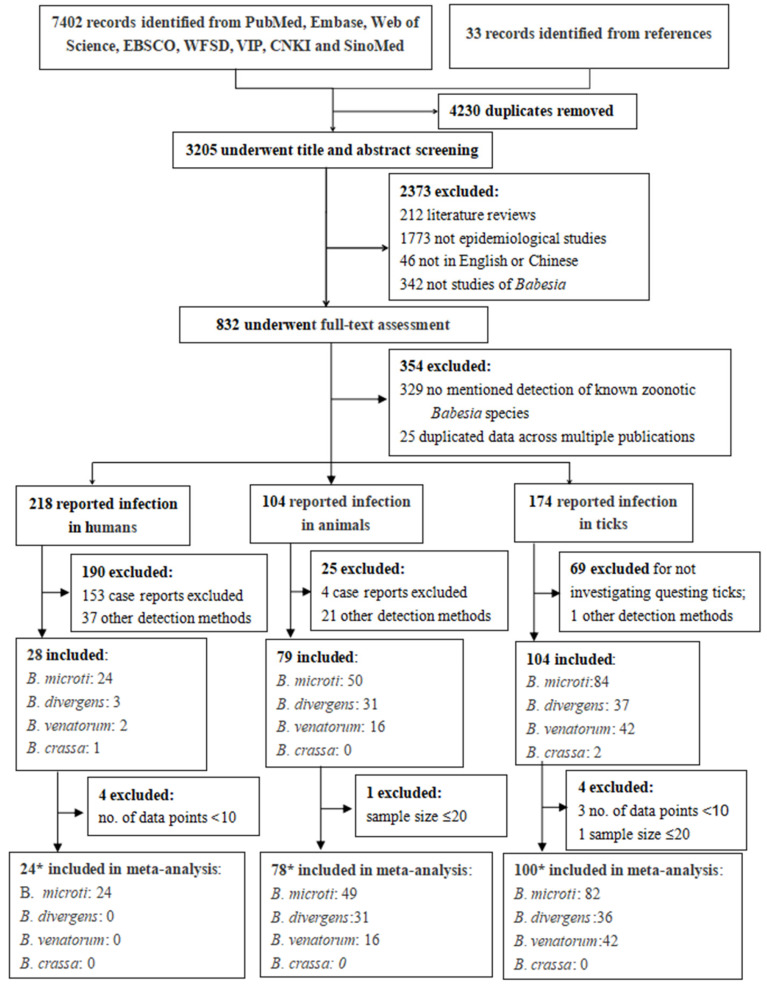
Flow diagram of study selection. * In the included studies, a total of 18 studies reported dual infection of humans, animals or ticks, of which four studies reported the infection of human and ticks [31,32,33,34], and 14 studies reported the infection of ticks and animals [35,36,37,38,39,40,41,42,43,44,45,46,47,48]. In addition, some studies reported more than one zoonotic *Babesia* infection.

Results of the literature search and selection are shown in Figure 1. Electronic database searches identified 7402 records. The review of relevant reference lists further identified 33 records. After removal of duplicates, 3205 articles were screened by titles and abstracts, resulting in 832 articles for full-text screening. After reviewing the full texts, 478 studies were found to be related to the infections of known zoonotic *Babesia* species, of which 218 reported infections in humans, 104 in animals and 174 in ticks. After excluding case reports, case series, articles merely reporting infection in infesting ticks, and studies using detecting methods other than nucleic acid test, 28 articles were left for the systematic review of human infection, 78 for animal infection and 104 for infection in ticks.

Among the 28 nucleic acid prevalence surveys in humans, 24 studies reported detection of *B. microti* infection, 3 reported *B. divergens* infection, 2 reported *B. venatorum* infection and 1 reported *B. crassa* infection. In the bias risk assessment, both assessors rated 17 of the studies to be of moderate quality, and 11 were of low quality (Table S1). Among the studies reporting nucleic acid prevalence in animals, 49 studies reported detection of *B. microti* infection, 31 reported *B. divergens* infection and 16 reported *B. venatorum* infection. Among the studies reporting nucleic acid prevalence in ticks, 84 studies reported detection of *B. microti* infection, 37 reported *B. divergens* infection, 42 reported *B. venatorum* infection and 2 reported *B. crassa* infection.

The basic characteristics and data extracted from the included studies for humans, animals and ticks are shown in Tables S1–S3. Overall, *B. microti* is most widely distributed, and its infection in humans, animals and ticks was reported in all continents except Antarctica; *B. divergens* and *B. venatorum* infections were mainly reported in Europe and occasionally in Asia; and *B. crassa* was reported in China and Turkey (Tables S1–S3).

### 3.2. Prevalence of Zoonotic BABESIA in Humans

*B. microti* in humans. Overall, 24 studies containing 852,344 blood samples were included in the meta-analysis to estimate the pooled nucleic acid prevalence of *B. microti* in humans.

The nucleic acid prevalence of *B. microti* in humans varied from 0.00% to 45.16%. The pooled prevalence was estimated to be 1.93% (95% CI: 0.32–4.69%), and the heterogeneity across the studies was high (I^2^ = 98.4%, *p* < 0.001, Table 1; forest plot is shown in Figure S1a). Subgroup analysis showed that type of population (R^2^ = 34.34%, QM = 12.24, *p* < 0.001), continent in which the study was performed (R^2^ = 29.06%, QM = 12.93, *p* = 0.012) and exact detecting method (R^2^ = 25.34%, QM = 9.02, *p* = 0.011) could explain the heterogeneity significantly (Table 1). When stratified by type of population, the pooled nucleic acid prevalence of *B. microti* was higher in high-risk populations (7.92% (95% CI: 3.14–14.49%)) than in general populations (0.30% (95% CI: 0.00–1.82%)); when stratified by continent, the pooled prevalence was highest in Asia (7.43%, 95% CI: 2.89–13.72%) and lowest in North America (0.06%, 95% CI: 0.00–1.59%); when stratified by exact detecting method, the pooled prevalence of nested PCR was highest (7.65% (95% CI: 2.58–14.96%)) compared with PCR (0.85% (95% CI: 0.00–3.92%)) and real-time PCR (0.10% (95% CI: 0.00–2.71%)). Results of multivariable meta-regression analysis showed that type of population, continent and detecting method could explain 39.33% of the heterogeneity (QM = 21.09, *p* = 0.004; Table S4).

*B. divergens* in humans. In general, three studies including 1757 samples reported nucleic acid prevalence of *B. divergens* in humans. One reported a prevalence of 0.48% in a high-risk population in the Netherlands [32], and the other two reported a prevalence of 0.53% in a general population and 1.33% in a high-risk population in China [49,50] (Table S1).

Other *Babesia* species in humans. Only China has reported human infections of *B. venatorum*. The reported nucleic acid prevalence was 1.65% in people with tick bite history from Northeast China (Table S1) [31].

All human infections of *B. duncani* were reported in the US until recently, but no prevalence survey was carried out using nucleic acid tests [51]. South Korea reported a new zoonotic *Babesia* species named *Babesia* sp. KO1 in 2007 [52]. Human infections of *Babesia* sp. XXB/HangZhou and *Babesia* sp. TW1 were separately reported in Zhejiang and Taiwan of China [53,54]. Two cases of *B. crassa* have been reported in Europe [55,56], and a prevalence survey reported a nucleic acid prevalence of 5.15% in people with tick bite history in China [57].

### 3.3. Prevalence of Zoonotic Babesia in Animals

*B. microti* in animals. Overall, 49 studies containing 32,958 animal blood or tissue samples were included in the meta-analysis to estimate the pooled nucleic acid prevalence of *B. microti* in animals, and the prevalence varied from 0.00% to 46.27% (Table S2).

The pooled nucleic acid prevalence was estimated to be 7.80% (95% CI: 5.25–10.77%) and the I^2^ value was 98.4% (*p* < 0.001, Table 2; forest plot in Figure S1b). Subgroup analysis showed that taxonomic order of animal host (R^2^ = 15.61%, QM = 18.36, *p* = 0.011), continent (R^2^ = 7.92%, QM = 8.32, *p* = 0.049) and sample type (R^2^ = 16.25%, QM = 13.86, *p* = 0.001) could explain the heterogeneity significantly. *B. microti* infections have been reported in many kinds of animals including Artiodactyla (ibex, cattle), Carnivora (cat, dog, bear), primates, shrews and even birds. The pooled prevalence was highest in Soricomorpha animals (14.81% (95% CI: 6.14–26.16%)) compared with other animals. When stratified by continent, the pooled prevalence was highest in North America (16.84% (95% CI: 7.60- 28.65)) compared with Asia (6.40% (95% CI: 2.99–10.91)), Europe (8.06% (95% CI 3.97–13.36)) and Africa (11.55% (95% CI: 0.10–35.75)). When stratified by sample type, the pooled prevalence of blood samples was higher (11.75% (95% CI: 8.07–15.99%)) than that of tissue samples (5.24% (95% CI: 1.96–9.83%)). The multivariable meta-regression model showed that taxonomy of animal host, continent, sample type and exact detecting method explained 41.81% of the total heterogeneity (QM = 57.41, *p* < 0.0001; Table S5).

*B. divergens* in animals. Overall, 31 studies containing 15,974 animal blood or tissue samples were included in the meta-analysis to estimate the pooled nucleic acid prevalence of *B. divergens* in animals, and the nucleic acid prevalence of *B. divergens* varied from 0.00% to 53.52% (Table S2). The pooled nucleic acid prevalence was estimated to be 2.12% (95% CI: 0.73–4.08%) and the I^2^ value was 95.4% (*p* < 0.001, Table 2; forest plot in Figure S1c). Most animal infections of *B. divergens* were reported in deer and cattle, and one study reported infection in rodents. Subgroup analysis and the meta-regression model showed that none of the moderators (taxonomy of animal host, continent of the study performed, sample type and exact detecting method) could explain the heterogeneity significantly.

Other zoonotic *Babesia* species in animals. *B. venatorum* infections have only been reported in Artiodactyla (deer, chamois, ibex) from Europe. In total, 17 studies reported nucleic acid prevalence, and the pooled prevalence was 1.42% (95% CI: 0.30–3.16) (Table 2; forest plots in Figure S1d).

*B. duncani* and *B. crassa* infection in animals has not been reported so far.

### 3.4. Prevalence of Zoonotic Babesia in Questing Ticks

*B. microti* in questing ticks. Overall, 82 studies reported *B. microti* infection in questing nymphs or adult ticks, and the prevalence varied from 0.00% to 58.33% (Table S3). Most of the infections in questing ticks were reported in *Ixodes* spp. However, *Haemaphysalis* spp., *Rhipicephalus* spp. and *Dermacentor* spp. were also potential vectors of *B. microti*. The pooled nucleic acid prevalence was estimated to be 2.36% (95% CI: 1.55–3.32%), and the I^2^ value was 97.7% (*p* < 0.001; Table 3; forest plot in Figure S1e). Subgroup analysis showed that none of the moderators (tick species, instar, continent and exact detecting method) could explain the heterogeneity significantly (Table 3). However, the multivariable meta-regression model showed that tick species, instar and detecting method explained 26.24% of the total heterogeneity (QM = 29.35, *p* < 0.001; Table S6).

Interestingly, two studies reported *B. microti* infection in questing larvae of *I. ricinus*, indicating the potential of trans-ovarial transmission by ticks [58,59].

*B. divergens* in questing ticks. Overall, 36 studies reported *B. divergens* infection in questing nymphs or adult ticks, and the prevalence varied from 0.00% to 4.29% (Table S3). Most *B. divergens* infections were reported in *I. ricinus* and *I. persulcatus* ticks. Moreover, infections in *Hae. longicornis* and *Hae. concinna* ticks were also reported. The pooled nucleic acid prevalence was estimated to be 0.25% (95% CI: 0.09–0.45%), and the I^2^ value was 90.57% (*p* < 0.001; Table 3; forest plot in Figure S1f). Subgroup analysis and the multivariable meta-regression model showed that instar could explain the heterogeneity significantly (R^2^ = 18.68%, QM = 10.69, *p* = 0.0048, Table 3).

*B. venatorum* in questing ticks. Overall, 42 studies reported *B. venatorum* infection in questing nymphs or adult ticks, and the prevalence varied from 0.00% to 7.69% (Table S3). Most infections in questing ticks were from *I. ricinus* and *I. persulcatus*, and infection in *Hae. concinna* was also reported. The pooled prevalence was estimated to be 0.43% (95% CI: 0.29–0.61%), and the I^2^ value was 81.1% (*p* < 0.001, Table 3, forest plot in Figure S1g). Subgroup analysis and the multivariable meta-regression model showed that tick species could explain the heterogeneity significantly (R^2^ = 20.78%, QM = 18.18, *p* = 0.0058, Table 3). The prevalence was higher in *I. ricinus* (0.50%, 95% CI: 0.34–0.68%) and *I. persulcatus* (0.53%, 95% CI: 0.26–0.88%) ticks than that in *Haemaphysalis spp.* Ticks (0.01%, 95% CI: 0.00–0.23%).

Moreover, one study reported *B. venatorum* infection in questing larvae of *I. ricinus*, indicating the potential of trans-ovarial transmission by ticks [60].

Other zoonotic *Babesia* species in questing ticks. Two studies reported prevalence of *B. crassa* in questing ticks. One reported a prevalence of 1.01% in questing adults of *Hae. parva* in Turkey [61] and the other reported a prevalence of 0.31% in *I. persulcatus* and 0.40% in *H. concinna* in China [57] (Table S3). *B. duncani* and *B. microti-like* infection in questing ticks has not been reported so far.

### 3.5. Publication Bias and Sensitivity Analysis

Funnel plots asymmetry and the result of Egger’s test revealed the existence of publication bias (Figure S2). Results of sensitive analysis showed that neither outlier data point removal nor data points with small sample sizes changed the pooled nucleic acid prevalence estimate significantly (95% CI overlapped, Table S7).

## 4. Discussion

Zoonotic *Babesia* spp. infections are an emerging public health threat globally [57]. Geographical distribution, animal reservoirs and tick vectors vary greatly across *Babesia* species [62]. Therefore, summarizing global data according to different species enables us to better understand the situation of these pathogens. In this systematic review and meta-analysis, we reported the global nucleic acid prevalence of different zoonotic *Babesia* species in humans, animals and ticks. To the best of our knowledge, this is the first systematic review to summarize nucleic acid prevalence of zoonotic *Babesia* in humans, animals and ticks according to various species.

*B. microti* is the most widely distributed *Babesia* species and its infection in humans, animals and ticks was reported worldwide [63]. Consistent with our knowledge, the results of subgroup analysis showed that pooled prevalence of *B. microti* was higher in high-risk populations than that in general populations (Table 1). Pooled estimates of human *B. microti* infection also varied across geographic regions with the highest in Asia and the lowest in North America (Table 1). However, pooled prevalence estimates of *B. microti* in animals (Table 2 and Table S5) and ticks (Table S6) were highest in North America. The conflicting results may be attributed to the type of study population in human studies. For example, all 10 of the data points of humans from North America targeted general populations (blood donors), while 7 out of 10 data points from Asia targeted high-risk populations. Moreover, when stratified by exact detecting method, the pooled estimate of human *B. microti* infection was higher using nested PCR than PCR or real-time PCR (Table 1). These variations may be attributed to the sensitivity difference across detecting methods [64,65]. Considering that most of the nucleic acid prevalence surveys used PCR testing (10 out of 24 studies), prevalence of *B. microti* in humans might be underestimated.

Although rodents (mainly referring to mice) were considered to be the primary intermediate animal host reservoir of *B. microti* [66,67], its infection has been reported in many kinds of animals including Carnivora (cat, dog, bear), Soricomorpha, Artiodactyla (ibex, cattle), primates, shrews and even birds (Table 2 and Table S2). The wide range of animal hosts and high prevalence in animals might contribute to the worldwide distribution of *B. microti*. However, animals other than rodents might be accidental hosts of *B. microti*, and whether they play roles in the transmission of this parasite is still needed to be elucidated [68,69] [70]. *B. microti* is considered to be mainly transmitted by *Ixodes* ticks [71]. However, ticks of other genera including *Haemaphysalis*, *Rhipicephalus* and *Dermacentor* are also potential vectors. This might be another possible reason for the wide distribution of this parasite.

*B. divergens* infections in humans, animals and ticks have only been reported in Europe and Asia. The pooled estimates of *B. divergens* infection in humans, animals and ticks were lower than those of *B. microti* (Table 1, Table 2 and Table 3). Animals of Artiodactyla (deer or bovine) are primary animal reservoirs, and infections in mice have also been reported. Most *B. divergens* infections were reported in *I. ricinus* and *I. persulcatus* ticks, and infections in *H. longicornis* and *H. concinna* ticks were occasionally reported. The lower prevalence and limited number of animal reservoirs and tick vectors might explain the limited distribution of *B. divergens*.

Interestingly, geographical distribution, animal reservoirs and possible transmission vectors of *B. venatorum* were exactly similar to those of *B. divergens*. For example, similar with *B. divergens*, infections of *B. venatorum* have only been reported in Europe and Asia; animals of Artiodactyla are known primary reservoirs, and *I. ricinus*, *I. persulcatus* and *H. concinna* are known vectors (Table 2 and Table 3). These phenomena may be attributable to their close kinship. Phylogenetic analysis based on comparing the complete 18S rRNA gene sequence showed that *B. venatorum* is closely related to *B. divergens* [72]. *B. venatorum* has also been identified in splenectomized humans infected with *B. divergens* [73].

In the present study, studies from many centers were pooled for a relatively large sample size to summarize the global nucleic acid prevalence of different zoonotic *Babesia* species in humans, animals and ticks. However, the several limitations of our study should be considered. First, most of the data came from small-scale surveys (such as a park), and articles published in languages other than English and Chinese were not included in this study, which does not represent all countries. Moreover, studies were unevenly distributed all over the world. This may cause bias in the pooled global estimates of zoonotic *Babesia* infections. However, these are the best available global estimates currently. Second, significant heterogeneity was detected across studies. Although some subgroup analyses were conducted to identify the sources of heterogeneity, many unmeasured moderators have impacts on the results. For example, the primer sets used in the included studies varied, and the sensitivity of different primer sets was unclear [74]. Finally, similar with other meta-analyses on tick-borne infections [11,75,76], publication bias exists in the present study, which may distort the estimates of prevalence, so results should be interpreted with caution.

## 5. Conclusions

Geographical distribution, animal reservoirs and tick vectors vary greatly across *Babesia* species. *B. microti* is the most prevalent and most widely distributed zoonotic *Babesia* species globally. The wide range of suitable animal reservoirs and potential transmission vectors and high prevalence in animals and ticks may contribute to the worldwide distribution of *B. microti*. *B. divergens* and *B. venatorum* were relatively less prevalent and were reported in quite limited areas compared with *B. microti*. Moreover, geographical distribution, animal reservoirs and possible transmission vectors of *B. divergens* and *B. venatorum* were similar.

## Figures and Tables

**Table 1:** Estimates of pooled nucleic acid prevalence and subgroup analysis of zoonotic *Babesia* in humans.

	No. of Data Points	Sample Size	No. of Positives	Pooled Prevalence, % (95% CI)	I^2^, % (95% CI)	R^2^, %	QM (p)	QE (p)
*B. microti*	24	852,344	933	1.93 (0.32; 4.69)	98.4 (98.2; 98.7)			
Type of population						34.34	12.24 (0.0005)	1215.40 (<0.0001)
General population	15	849,480	776	0.30 (0.00; 1.82)	98.4			
High-risk population	9	2864	157	7.92 (3.14; 14.49)	97.6			
Continent						29.06	12.93 (0.0116)	1041.26 (<0.0001)
Asia	10	4790	203	7.43 (2.89; 13.72)	97.3			
North America	10	845,911	713	0.06 (0.00; 1.59)	98.7			
Europe	2	1174	6	0.33 (0.00; 7.91)	89.2			
South America	1	271	9	3.32 (0.00; 23.29)	NE			
Africa	1	198	2	1.01 (0.00; 17.27)	NE			
Testing method						25.34	9.02 (0.0110)	1033.13 (<0.0001)
PCR	9	226,805	145	0.85 (0.00; 3.92)	91.2			
Nested PCR	8	3742	189	7.65 (2.58; 14.96)	97.0			
Real-time PCR	7	621,797	599	0.10 (0.00; 2.71)	99.3			

NE: not estimated; R^2^ is the proportion of true heterogeneity that can be explained by the moderator, the QE statistic and its *p* value show the significance of residual heterogeneity that is unaccounted for by the moderator, and the QM statistic and its *p* value show whether the moderator is statistically significant in explaining heterogeneity.

**Table 2:** Estimates of pooled nucleic acid prevalence and subgroup analysis of zoonotic *Babesia* in animals.

	No. of Data Points	Sample Size	No. of Positives	Pooled Prevalence, % (95% CI)	I^2^, % (95% CI)	R^2^, %	QM (p)	QE (p)
*B. microti*	66	32958	2815	7.80 (5.25; 10.77)	98.4 (98.3; 98.6)			
Taxonomy of animal host						15.61	18.36 (0.0105)	3126.73 (<0.0001)
Rodent	39	24,466	2344	10.13 (6.76; 14.07)	98.70			
Carnivora	5	1673	33	1.98 (0.00; 9.50)	68.74			
Soricomorpha	8	1132	259	14.81 (6.14; 26.16)	95.40			
Primate	4	2354	150	8.05 (0.92; 20.97)	93.50			
Artiodactyla	5	2723	2	0.02 (0.00; 4.11)	36.10			
Chiroptera	1	203	9	4.43 (0.00; 31.17)	NE			
Passeriformes	2	121	18	9.10 (0.00; 30.97)	96.70			
Lagomorpha	2	286	0	0.00 (0.00; 9.59)	NE			
Continent						7.92	8.32 (0.0490)	3835.07 (<0.0001)
North America	9	1820	315	16.84 (7.60; 28.65)	98.10			
Europe	26	14,275	1342	8.06 (3.97; 13.36)	99.20			
Asia	29	16,535	1122	6.40 (2.99; 10.91)	96.70			
Africa	2	328	36	11.55 (0.10; 35.75)	95.50			
Sample type						16.25	13.86 (0.0010)	2710.05 (<0.0001)
Blood	38	15,608	2266	11.75 (8.07; 15.99)	98.40			
Tissue	20	10,248	429	5.24 (1.96; 9.83)	94.10			
Blood + Tissue	8	7102	120	0.79 (0.00; 4.94)	96.50			
Nucleic acid testing method						3.64	4.46 (0.1078)	4104.60 (<0.0001)
PCR	29	10,938	730	4.83 (2.02; 8.65)	96.50			
Nested PCR	26	15,000	1343	11.16 (6.64; 16.64)	99.50			
Real-time PCR	11	7020	742	9.01 (6.64; 16.64)	98.20			
*B. divergens*	35	15,974	381	2.12 (0.73; 4.08)	95.4 (94.4; 96.3)			
Taxonomy of animal host						0.52	4.20 (0.3790)	556.88 (<0.0001)
Artiodactyla	27	12,219	379	3.16 (1.26; 5.77)	95.30			
Rodent	5	2647	2	0.07 (0.00; 3.91)	56.50			
Carnivora	1	812	0	0.00 (0.00; 10.20)	NE			
Lagomorpha	1	238	0	0.00 (0.00; 11.30)	NE			
Passeriformes	1	58	0	0.00 (0.00; 14.39)	NE			
Continent						0.00	0.22 (0.8962)	635.06 (<0.0001)
Europe	27	13,014	228	2.16 (0.61; 4.47)	94.70			
North America	1	33	0	0.00 (0.00; 17.57)	NE			
Asia	7	2927	153	2.44 (0.00; 8.21)	95.70			
Nucleic acid testing method						0.00	0.59 (0.7434)	668.94 (<0.0001)
PCR	22	9898	286	1.94 (0.33; 4.52)	94.70			
Nested PCR	5	900	26	3.92 (0.05; 11.92)	91.10			
Real-time PCR	8	5176	69	1.76 (0.00; 6.15)	96.90			
Sample type						4.99	3.64 (0.1621)	633.72 (<0.0001)
Blood	24	9250	319	3.17 (1.19; 5.91)	95.80			
Tissue	5	1683	31	1.32 (0.00; 7.08)	81.90			
Blood + Tissue	6	5041	31	0.17 (0.00; 3.06)	92.00			
*B. venatorum*	22	9186	120	1.42 (0.30; 3.16)	92.9 (90.6; 94.7)			
Taxonomy of animal host						24.60	9.97 (0.0410)	198.97 (<0.0001)
Artiodactyla	14	5205	120	3.26 (1.32; 5.92)	93.40			
Carnivora	3	1121	0	0.00 (0.00; 2.39)	NE			
Lagomorpha	1	238	0	0.00 (0.00; 6.21)	NE			
Rodent	3	2564	0	0.00 (0.00; 2.01)	NE			
Passeriformes	1	58	0	0.00 (0.00; 8.90)	NE			
Continent						0.00	0.73 (0.3929)	292.72 (<0.0001)
Asia	1	408	0	0.00 (0.00; 7.44)	0.00			
Europe	21	8778	120	1.56 (0.34; 3.43)	93.20			
Exact testing method						0.00	0.89 (0.6423)	278.08 (<0.0001)
PCR	11	3566	74	2.20 (0.32; 5.36)	93.60			
Real-time PCR	8	5070	42	0.92 (0.00; 3.71)	93.70			
Nested PCR	3	550	4	0.53 (0.00; 6.18)	82.20			
Sample						7.11	3.30 (0.1923)	213.88 (<0.0001)
Blood	10	2370	72	2.71 (0.52; 6.18)	92.90			
Tissue	6	1775	37	1.50 (0.00; 5.15)	91.90			
Blood + Tissue	6	5041	11	0.23 (0.00; 2.39)	80.50			
*B. microti*-like	10	6487	471	42.14 (20.65; 65.26)	99.5 (99.4; 99.6)			
Taxonomy of animal host						0.00	0.01 (0.9349)	1629.65 (<0.0001)
Carnivora	8	6437	449	41.70 (17.13; 68.62)	99.60			
Rodent	2	50	22	44.14 (1.59; 93.26)	0.00			
Continent						0.00	0.33 (0.8467)	707.96 (<0.0001)
Asia	3	74	24	30.46 (0.20; 77.29)	81.80			
North America	3	5294	88	48.00 (8.35; 89.23)	99.40			
Europe	4	1119	359	46.32 (11.44; 83.40)	99.10			
Exact testing method						0.00	0.58 (0.4481)	936.22 (<0.0001)
PCR	4	5385	151	53.38 (18.37; 86.62)	99.50			
Nested PCR	6	1102	320	34.64 (9.42; 65.40)	98.20			
Sample						0.00	0.15 (0.7006)	957.17 (<0.0001)
Blood	7	6187	275	39.07 (13.77; 67.87)	99.40			
Tissue	3	300	194	49.48 (10.38; 88.96)	92.70			

NE: not estimated; R^2^ is the proportion of true heterogeneity that can be explained by the moderator, the QE statistic and its *p* value show the significance of residual heterogeneity that is unaccounted for by the moderator, and the QM statistic and its *p* value show whether the moderator is statistically significant in explaining heterogeneity.

**Table 3:** Estimates of pooled nucleic acid prevalence and subgroup analysis of zoonotic *Babesia* in questing ticks.

	No. of Data Points	Sample Size	No. of Positives	Pooled Prevalence, % (95% CI)	I^2^, % (95% CI)	R^2^, %	QM (p)	QE (p)
*B. microti*	130	117134	2311	2.30 (1.59; 3.13)	97.1 (96.8; 97.4)			
Tick species						0.38	11.13 (0.4328)	3511.71 (<0.0001)
*Ixodes ricinus*	63	66,405	914	2.45 (1.43; 3.71)	97.20			
*Ixodes scapulans*	27	26,846	1005	4.22 (2.29; 6.67)	96.10			
*Ixodes persulcatus*	17	15,373	189	1.29 (0.16; 3.29)	95.90			
*Ixodes ovatus*	1	789	38	4.82 (0.00; 22.47)	NE			
*Haemaphysalis longicornis*	4	2678	113	3.21 (0.11; 9.75)	98.80			
*Haemaphysalis concinna*	6	2624	3	0.07 (0.00; 2.33)	46.80			
*Haemaphys alisqinghaiensis*	1	34	0	0.00 (0.00;14.30)	NE			
*Dermacentor reticulatus*	4	1339	38	2.12 (0.00; 8.10)	82.40			
*Dermacentor nuttalli*	3	219	0	0.00 (0.00; 4.93)	0.00			
*Rhipicephalus turanicus*	1	85	1	1.18 (0.00; 16.25)	NE			
*Rhipicephalus microplus*	2	372	6	1.43 (0.00; 10.11)	59.10			
*Rhipicephalus sanguineus*	1	370	4	1.08 (0.00; 13.97)	NE			
Genus of ticks						0.02	3.38 (0.3367)	4440.02 (<0.0001)
*Ixodes spp.*	108	109,413	2146	2.65 (1.82; 3.61)	97.40			
*Haemaphysalis spp.*	11	5336	116	0.73 (0.00; 3.04)	97.00			
*Dermacentor spp.*	7	1558	38	0.88 (0.00; 4.66)	77.50			
*Rhipicephalus spp.*	4	827	11	1.26 (0.00; 6.60)	0.00			
Instar						0.56	2.77 (0.2503)	4328.21 (<0.0001)
adult	65	41,781	1019	2.04 (1.12; 3.18)	95.80			
nymph	43	52,223	820	2.07 (1.00; 3.48)	97.40			
not distinguished	22	23,130	472	3.83 (1.75; 6.58)	98.20			
Continent						1.91	4.14 (0.1265)	3713.68 (<0.0001)
North America	28	27,211	1005	3.95 (2.13; 6.27)	96.20			
Europe	78	73,157	991	2.08 (1.23; 3.12)	96.60			
Asia	24	16,766	315	1.40 (0.28; 3.15)	96.90			
Exact detecting method						0.00	0.55 (0.7593)	4412.19 (<0.0001)
Nested PCR	44	26,621	555	2.73 (1.47; 4.34)	96.50			
Real-time PCR	25	34,091	581	2.00 (0.60; 4.03)	98.10			
PCR	61	56,422	1175	2.14 (1.18; 3.34)	96.90			
*B. divergens*	59	87,015	212	0.16 (0.05; 0.32)	88.2 (85.6; 90.4)			
Tick species						0.00	3.18 (0.8683)	464.76 (<0.0001)
*Ixodes ricinus*	37	66,338	151	0.16 (0.02; 0.38)	91.00			
*Ixodes persulcatus*	11	13,585	55	0.36 (0.05; 0.86)	89.80			
*Haemaphysalis concinna*	6	2624	3	0.08 (0.00; 0.64)	NE			
*Haemaphysalis longicornis*	1	390	3	0.77 (0.00; 3.87)	NE			
*Ixodes ovatus*	1	595	0	0.00 (0.00; 1.42)	NE			
*Ixodes scapularis*	1	2858	0	0.00 (0.00; 1.09)	NE			
*Dermacentor reticulatus*	1	582	0	0.00 (0.00; 1.43)	NE			
*Dermacentor nuttalli*	1	43	0	0.00 (0.00; 5.30)	NE			
Genus of ticks						0.00	0.41 (0.8149)	491.71 (<0.0001)
*Ixodes spp.*	50	83,376	206	0.18 (0.05; 0.37)	89.80			
*Haemaphysalis spp.*	7	3014	6	0.14 (0.00; 0.68)	34.40			
*Dermacentor spp.*	2	625	0	0.00 (0.00; 0.94)	NE			
Continent						0.00	1.13 (0.5688)	476.79 (<0.0001)
Asia	13	13,437	51	0.30 (0.04; 0.72)	88.30			
Europe	45	70,720	161	0.14 (0.02; 0.32)	88.30			
North America	1	2858	0	0.00 (0.00; 1.05)	NE			
Instar						18.68	10.69 (0.0048)	484.91 (<0.0001)
adult	30	26,087	21	0.07 (0.00; 0.25)	78.10			
nymph	16	24,826	18	0.04 (0.00; 0.24)	8.20			
not distinguished	13	36,102	133	0.73 (0.33; 1.25)	96.40			
Exact detecting method						6.73	5.88 (0.0528)	426.92 (<0.0001)
Real-time PCR	8	33,746	12	0.03 (0.00; 0.34)	48.90			
PCR	35	41,751	180	0.28 (0.10; 0.54)	91.40			
Nested PCR	16	11,518	20	0.06 (0.00; 0.33)	4.60			
*B. venatorum*	71	91,727	601	0.39 (0.26; 0.54)	77.0 (71.2;81.6)			
Tick species						20.78	18.18 (0.0058)	256.50 (<0.0001)
*Ixodes ricinus*	47	73,656	500	0.50 (0.34; 0.68)	76.50			
*Ixodes persulcatus*	13	13,712	100	0.53 (0.26; 0.88)	79.70			
*Haemaphysalis concinna*	5	2460	1	0.01 (0.00; 0.28)	0.00			
*Dermacentor nuttalli*	2	168	0	0.00 (0.00; 1.29)	0.00			
*Dermacentor reticulatus*	2	746	0	0.00 (0.00; 0.49)	0.00			
*Haemaphysalis longicornis*	1	390	0	0.00 (0.00; 0.85)	NE			
*Ixodes ovatus*	1	595	0	0.00 (0.00; 0.69)	NE			
Genus of ticks						21.91	14.86 (0.0006)	265.51 (<0.0001)
*Ixodes spp.*	61	87,963	600	0.49 (0.35; 0.64)	77.20			
*Haemaphysalis spp.*	6	2850	1	0.01 (0.00; 0.23)	0.00			
*Dermacentor spp.*	4	914	0	0.00 (0.00; 0.27)	0.00			
Continent						2.16	2.17 (0.1404)	299.68 (<0.0001)
Asia	14	13,164	75	0.25 (0.06; 0.54)	80.00			
Europe	57	78,563	526	0.43 (0.28; 0.60)	76.10			
Instar						0.00	2.45 (0.2936)	280.71 (<0.0001)
adult	37	27,082	169	0.35 (0.19; 0.55)	71.00			
nymph	23	26,096	133	0.34 (0.14; 0.61)	76.10			
not distinguished	11	38,549	299	0.63 (0.28; 1.09)	84.50			
Exact detecting method						0.00	0.34 (0.8440)	302.28 (<0.0001)
Nested PCR	21	16,672	124	0.38 (0.16; 0.66)	75.30			
Real-time PCR	14	35,652	228	0.48 (0.19; 0.86)	85.70			
PCR	36	39,403	249	0.37 (0.19; 0.58)	73.10			

NE: not estimated; R^2^ is the proportion of true heterogeneity that can be explained by the moderator, the QE statistic and its *p* value show the significance of residual heterogeneity that is unaccounted for by the moderator, and the QM statistic and its *p* value show whether the moderator is statistically significant in explaining heterogeneity.

## Data Availability

The data presented in this study are available on request from the corresponding author.

## Supplementary Materials

The following supporting information can be downloaded at: https://www.mdpi.com/article/10.3390/tropicalmed8030132/s1. Availability of data and materials: The data that supports the findings of this study are available in the supplementary material of this article. Additional files: Table S1. Publications for full-text screen of zoonotic *Babesia* in humans. Table S2. Publications for full-text screen of zoonotic *Babesia* in animals. Table S3. Publications for full-text screen of zoonotic *Babesia* in ticks. Table S4. Multivariable meta-regression analyses for moderator on nucleic acid prevalence estimates of zoonotic *Babesia* in humans. Table S5. Multivariable meta-regression analyses for moderators on nucleic acid prevalence estimates of zoonotic *Babesia* in animals. Table S6. Multivariable meta-regression analyses for moderators on nucleic acid prevalence estimates of zoonotic *Babesia* in questing ticks. Table S7. Sensitivity analysis of the pooled nucleic acid prevalence of zoonotic *Babesia* in humans, animals and ticks. Additional file 8: Figure S1. Forest plots of nucleic acid prevalence of each zoonotic *Babesia* species in humans, animals, and ticks (Main Analyses) (a) *B. microti* in humans; (b) *B. microti* in animals; (c) *B. divergens* in animals; (d) *B. venatorum* in animals; (e) *B. microti* in questing ticks; (f) *B. divergens* in questing ticks; (g) *B. venatorum* in questing ticks. Additional file 9: Figure S2. Funnel plot for assessing publication bias in studies reporting nucleic acid prevalence of each zoonotic *Babesia* species in humans, animals, and ticks (a) *B. microti* in humans; (b) *B. microti* in animals; (c) *B. divergens* in animals; (d) *B. venatorum* in animals; (e) *B. microti* in questing ticks; (f) *B. divergens* in questing ticks; (g) *B. venatorum* in questing ticks.

## Abbreviations

C.I.	Confidence Interval
PCR	Polymerase Chain Reaction

## References

[1] Remesar S., Díaz P., Prieto A., Markina F., Díaz Cao J.M., López-Lorenzo G., Fernández G., López C.M., Panadero R., Díez-Baños P. (2019). Prevalence and distribution of *Babesia* and *Theileria* species in roe deer from Spain. Int. J. Parasitol. Parasites Wildl..

[2] Antunes S., Rosa C., Couto J., Ferrolho J., Domingos A. (2017). Deciphering *Babesia*-Vector Interactions. Front. Cell. Infect. Microbiol..

[3] Elsworth B., Duraisingh M.T. (2021). A framework for signaling throughout the life cycle of *Babesia* species. Mol. Microbiol..

[4] Krause P.J. (2019). Human babesiosis. Int. J. Parasitol..

[5] Wang F., Jiang J.F., Tian J., Du C.H. (2020). Clinical characteristics, diagnosis and treatment of human babesiosis: A review. Zhongguo xue xi chong bing fang zhi za zhi = Chin. J. Schistosomiasis Control..

[6] Laha R., Das M., Sen A. (2015). Morphology, epidemiology, and phylogeny of *Babesia*: An overview. Trop. Parasitol..

[7] Nardini R., Bartolomé Del Pino L.E., Cersini A., Manna G., Viola M.R., Antognetti V., Autorino G.L., Scicluna M.T. (2021). Comparison of PCR-based methods for the detection of *Babesia caballi* and *Theileria equi* in field samples collected in Central Italy. Parasitol. Res..

[8] Gabrielli S., Calderini P., Cassini R., Galuppi R., Tampieri M.P., Pietrobelli M., Cancrini G. (2014). Human exposure to piroplasms in Central and Northern Italy. Vet. Ital..

[9] Zanet S., Trisciuoglio A., Bottero E., de Mera I.G., Gortazar C., Carpignano M.G., Ferroglio E. (2014). Piroplasmosis in wildlife: *Babesia* and *Theileria* affecting free-ranging ungulates and carnivores in the Italian Alps. Parasites Vectors.

[10] Karshima S.N., Karshima M.N., Ahmed M.I. (2022). Global meta-analysis on *Babesia* infections in human population: Prevalence, distribution and species diversity. Pathog. Glob. Health.

[11] Karshima S.N., Karshima M.N., Ahmed M.I. (2022). Infection rates, species diversity, and distribution of zoonotic *Babesia* parasites in ticks: A global systematic review and meta-analysis. Parasitol. Res..

[12] Karshima S.N., Karshima M.N., Ahmed M.I. (2021). Animal reservoirs of zoonotic *Babesia* species: A global systematic review and meta-analysis of their prevalence, distribution and species diversity. Vet. Parasitol..

[13] Onyiche T.E., Răileanu C., Fischer S., Silaghi C. (2021). Global Distribution of *Babesia* Species in Questing Ticks: A Systematic Review and Meta-Analysis Based on Published Literature. Pathogens.

[14] Fischer C., Jo W.K., Haage V., Moreira-Soto A., de Oliveira Filho E.F., Drexler J.F. (2021). Challenges towards serologic diagnostics of emerging arboviruses. Clin. Microbiol. Infect.: Off. Publ. Eur. Soc. Clin. Microbiol. Infect. Dis..

[15] Yu A.C., Vatcher G., Yue X., Dong Y., Li M.H., Tam P.H., Tsang P.Y., Wong A.K., Hui M.H., Yang B. (2012). Nucleic acid-based diagnostics for infectious diseases in public health affairs. Front. Med..

[16] Overzier E., Pfister K., Herb I., Mahling M., Böck G.J., Silaghi C. (2013). Detection of tick-borne pathogens in roe deer (*Capreolus capreolus*), in questing ticks (*Ixodes ricinus*), and in ticks infesting roe deer in southern Germany. Ticks Tick-Borne Dis..

[17] Lai M.C., Kassee C., Besney R., Bonato S., Hull L., Mandy W., Szatmari P., Ameis S.H. (2019). Prevalence of co-occurring mental health diagnoses in the autism population: A systematic review and meta-analysis. Lancet Psychiatry.

[18] Farkas R., Takács N., Hornyák Á., Nachum-Biala Y., Hornok S., Baneth G. (2015). First report on *Babesia* cf. *microti* infection of red foxes (*Vulpes vulpes*) from Hungary. Parasites Vectors.

[19] Lee S., Hong Y., Chung D.I., Jang H.K., Goo Y.K., Xuan X. (2022). Evolutionary analysis of *Babesia vulpes* and *Babesia microti*-like parasites. Parasites Vectors.

[20] Sun X., Zhen X., Hu X., Li Y., Gu S., Gu Y., Dong H. (2019). Osteoarthritis in the Middle-Aged and Elderly in China: Prevalence and Influencing Factors. Int. J. Environ. Res. Public Health.

[21] Hoy D., Brooks P., Woolf A., Blyth F., March L., Bain C., Baker P., Smith E., Buchbinder R. (2012). Assessing risk of bias in prevalence studies: Modification of an existing tool and evidence of interrater agreement. J. Clin. Epidemiol..

[22] Barendregt J.J., Doi S.A., Lee Y.Y., Norman R.E., Vos T. (2013). Meta-analysis of prevalence. J. Epidemiol. Community Health.

[23] Crisafulli S., Sultana J., Fontana A., Salvo F., Messina S., Trifirò G. (2020). Global epidemiology of Duchenne muscular dystrophy: An updated systematic review and meta-analysis. Orphanet J. Rare Dis..

[24] Melsen W.G., Bootsma M.C., Rovers M.M., Bonten M.J. (2014). The effects of clinical and statistical heterogeneity on the predictive values of results from meta-analyses. Clin. Microbiol. Infect.: Off. Publ. Eur. Soc. Clin. Microbiol. Infect. Dis..

[25] Kanters S. (2022). Fixed- and Random-Effects Models. Methods in Molecular Biology.

[26] Aubin J.M., Rekman J., Vandenbroucke-Menu F., Lapointe R., Fairfull-Smith R.J., Mimeault R., Balaa F.K., Martel G. (2013). Systematic review and meta-analysis of liver resection for metastatic melanoma. Br. J. Surg..

[27] Yong P.J., Matwani S., Brace C., Quaiattini A., Bedaiwy M.A., Albert A., Allaire C. (2020). Endometriosis and Ectopic Pregnancy: A Meta-analysis. J. Minim. Invasive Gynecol..

[28] Sha C.M., Lehrer E.J., Hwang C., Trifiletti D.M., Mackley H.B., Drabick J.J., Zaorsky N.G. (2020). Toxicity in combination immune checkpoint inhibitor and radiation therapy: A systematic review and meta-analysis. Radiother. Oncol.: J. Eur. Soc. Ther. Radiol. Oncol..

[29] Viechtbauer W., Cheung M.W. (2010). Outlier and influence diagnostics for meta-analysis. Res. Synth. Methods.

[30] Baujat B., Mahé C., Pignon J.P., Hill C. (2002). A graphical method for exploring heterogeneity in meta-analyses: Application to a meta-analysis of 65 trials. Stat. Med..

[31] Liu H.B., Wei R., Ni X.B., Zheng Y.C., Huo Q.B., Jiang B.G., Ma L., Jiang R.R., Lv J., Liu Y.X. (2019). The prevalence and clinical characteristics of tick-borne diseases at One Sentinel Hospital in Northeastern China. Parasitology.

[32] Jahfari S., Hofhuis A., Fonville M., van der Giessen J., van Pelt W., Sprong H. (2016). Molecular Detection of Tick-Borne Pathogens in Humans with Tick Bites and Erythema Migrans, in the Netherlands. PLoS Negl. Trop. Dis..

[33] Chen Z., Li H., Gao X., Bian A., Yan H., Kong D., Liu X. (2019). Human Babesiosis in China: A systematic review. Parasitol. Res..

[34] O’Brien S.F., Delage G., Scalia V., Lindsay R., Bernier F., Dubuc S., Germain M., Pilot G., Yi Q.L., Fearon M.A. (2016). Seroprevalence of *Babesia microti* infection in Canadian blood donors. Transfusion.

[35] Burkot T.R., Schneider B.S., Pieniazek N.J., Happ C.M., Rutherford J.S., Slemenda S.B., Hoffmeister E., Maupin G.O., Zeidner N.S. (2000). *Babesia microti* and *Borrelia bissettii* transmission by *Ixodes spinipalpis* ticks among prairie voles, *Microtus ochrogaster*, in Colorado. Parasitology.

[36] Hamšíková Z., Kazimírová M., Haruštiaková D., Mahríková L., Slovák M., Berthová L., Kocianová E., Schnittger L. (2016). *Babesia* spp. in ticks and wildlife in different habitat types of Slovakia. Parasites Vectors.

[37] Azagi T., Jaarsma R.I., Docters van Leeuwen A., Fonville M., Maas M., Franssen F.F.J., Kik M., Rijks J.M., Montizaan M.G., Groenevelt M. (2021). Circulation of *Babesia* Species and Their Exposure to Humans through *Ixodes ricinus*. Pathogens.

[38] Wang H., Yang J., Mukhtar M.U., Liu Z., Zhang M., Wang X. (2019). Molecular detection and identification of tick-borne bacteria and protozoans in goats and wild Siberian roe deer (*Capreolus pygargus*) from Heilongjiang Province, northeastern China. Parasites Vectors.

[39] Rar V.A., Epikhina T.I., Livanova N.N., Panov V.V. (2011). Genetic diversity of *Babesia* in *Ixodes persulcatus* and small mammals from North Ural and West Siberia, Russia. Parasitology.

[40] Rar V., Yakimenko V., Makenov M., Tikunov A., Epikhina T., Tancev A., Bobrova O., Tikunova N. (2016). High prevalence of *Babesia microti* ‘Munich’ type in small mammals from an *Ixodes persulcatus*/*Ixodes trianguliceps* sympatric area in the Omsk region, Russia. Parasitol. Res..

[41] Chen Z., Liu Q., Jiao F.C., Xu B.L., Zhou X.N. (2014). Detection of piroplasms infection in sheep, dogs and hedgehogs in Central China. Infect. Dis. Poverty.

[42] Jiang J., An H., Lee J.S., O’Guinn M.L., Kim H.C., Chong S.T., Zhang Y., Song D., Burrus R.G., Bao Y. (2018). Molecular characterization of *Haemaphysalis longicornis*-borne rickettsiae, Republic of Korea and China. Ticks Tick-Borne Dis..

[43] Jouglin M., Perez G., Butet A., Malandrin L., Bastian S. (2017). Low prevalence of zoonotic *Babesia* in small mammals and *Ixodes ricinus* in Brittany, France. Vet. Parasitol..

[44] Obiegala A., Pfeffer M., Pfister K., Karnath C., Silaghi C. (2015). Molecular examinations of *Babesia microti* in rodents and rodent-attached ticks from urban and sylvatic habitats in Germany. Ticks Tick-Borne Dis..

[45] Blaňarová L., Stanko M., Miklisová D., Víchová B., Mošanský L., Kraljik J., Bona M., Derdáková M. (2016). Presence of *Candidatus* Neoehrlichia mikurensis and *Babesia microti* in rodents and two tick species (*Ixodes ricinus* and *Ixodes trianguliceps*) in Slovakia. Ticks Tick-Borne Dis..

[46] Li L.H., Zhu D., Zhang C.C., Zhang Y., Zhou X.N. (2016). Experimental transmission of *Babesia microti* by *Rhipicephalus haemaphysaloides*. Parasites Vectors.

[47] Da Rold G., Ravagnan S., Soppelsa F., Porcellato E., Soppelsa M., Obber F., Citterio C.V., Carlin S., Danesi P., Montarsi F. (2018). Ticks are more suitable than red foxes for monitoring zoonotic tick-borne pathogens in northeastern Italy. Parasites Vectors.

[48] Georges K., Loria G.R., Riili S., Greco A., Caracappa S., Jongejan F., Sparagano O. (2001). Detection of haemoparasites in cattle by reverse line blot hybridisation with a note on the distribution of ticks in Sicily. Vet. Parasitol..

[49] Qi C., Zhou D., Liu J., Cheng Z., Zhang L., Wang L., Wang Z., Yang D., Wang S., Chai T. (2011). Detection of *Babesia divergens* using molecular methods in anemic patients in Shandong Province, China. Parasitol. Res..

[50] Wang J., Zhang S., Yang J., Liu J., Zhang D., Li Y., Luo J., Guan G., Yin H. (2019). *Babesia divergens* in human in Gansu province, China. Emerg. Microbes Infect..

[51] Prince H.E., Lapé-Nixon M., Patel H., Yeh C. (2010). Comparison of the *Babesia duncani* (WA1) IgG detection rates among clinical sera submitted to a reference laboratory for WA1 IgG testing and blood donor specimens from diverse geographic areas of the United States. Clin. Vaccine Immunol.: CVI.

[52] Kim J.Y., Cho S.H., Joo H.N., Tsuji M., Cho S.R., Park I.J., Chung G.T., Ju J.W., Cheun H.I., Lee H.W. (2007). First case of human babesiosis in Korea: Detection and characterization of a novel type of *Babesia* sp. (KO1) similar to ovine *Babesia*. J. Clin. Microbiol..

[53] Shih C.M., Liu L.P., Chung W.C., Ong S.J., Wang C.C. (1997). Human babesiosis in Taiwan: Asymptomatic infection with a *Babesia microti*-like organism in a Taiwanese woman. J. Clin. Microbiol..

[54] Man S.Q., Qiao K., Cui J., Feng M., Fu Y.F., Cheng X.J. (2016). A case of human infection with a novel *Babesia* species in China. Infect. Dis. Poverty.

[55] Strasek-Smrdel K., Korva M., Pal E., Rajter M., Skvarc M., Avsic-Zupanc T. (2020). Case of *Babesia crassa*-Like Infection, Slovenia, 2014. Emerg. Infect. Dis..

[56] Doderer-Lang C., Filisetti D., Badin J., Delale C., Clavier V., Brunet J., Gommenginger C., Abou-Bacar A., Pfaff A.W. (2022). *Babesia crassa*-Like Human Infection Indicating Need for Adapted PCR Diagnosis of Babesiosis, France. Emerg. Infect. Dis..

[57] Jia N., Zheng Y.C., Jiang J.F., Jiang R.R., Jiang B.G., Wei R., Liu H.B., Huo Q.B., Sun Y., Chu Y.L. (2018). Human Babesiosis Caused by a *Babesia crassa*-Like Pathogen: A Case Series. Clin. Infect. Dis.: Off. Publ. Infect. Dis. Soc. Am..

[58] Skotarczak B., Wodecka B., Cichocka A. (2002). Coexistence DNA of *Borrelia burgdorferi* sensu lato and *Babesia microti* in *Ixodes ricinus* ticks from north-western Poland. Ann. Agric. Environ. Med.: AAEM.

[59] Skotarczak B., Cichocka A. (2001). Isolation and amplification by polymerase chain reaction DNA of *Babesia microti* and *Babesia divergens* in ticks in Poland. Ann. Agric. Environ. Med.: AAEM.

[60] Schorn S., Pfister K., Reulen H., Mahling M., Silaghi C. (2011). Occurrence of *Babesia* spp., *Rickettsia* spp. and *Bartonella* spp. in *Ixodes ricinus* in Bavarian public parks, Germany. Parasites Vectors.

[61] Orkun Ö., Çakmak A., Nalbantoğlu S., Karaer Z. (2020). Turkey tick news: A molecular investigation into the presence of tick-borne pathogens in host-seeking ticks in Anatolia; Initial evidence of putative vectors and pathogens, and footsteps of a secretly rising vector tick, *Haemaphysalis parva*. Ticks Tick-Borne Dis..

[62] Hunfeld K.P., Brade V. (2004). Zoonotic *Babesia*: Possibly emerging pathogens to be considered for tick-infested humans in Central Europe. Int. J. Med. Microbiol.: IJMM.

[63] Kumar A., O’Bryan J., Krause P.J. (2021). The Global Emergence of Human Babesiosis. Pathogens.

[64] Au W.Y., Cheung P.P.H. (2021). Diagnostic performances of common nucleic acid tests for SARS-CoV-2 in hospitals and clinics: A systematic review and meta-analysis. Lancet Microbe.

[65] Kim E.J., Bauer C., Grevelding C.G., Quack T. (2013). Improved PCR/nested PCR approaches with increased sensitivity and specificity for the detection of pathogens in hard ticks. Ticks Tick-Borne Dis..

[66] Mardosaitė-Busaitienė D., Radzijevskaja J., Balčiauskas L., Paulauskas A. (2021). *Babesia microti* in Rodents from Different Habitats of Lithuania. Animals.

[67] Usluca S., Celebi B., Karasartova D., Gureser A.S., Matur F., Oktem M.A., Sozen M., Karatas A., Babur C., Mumcuoglu K.Y. (2019). Molecular Survey of *Babesia microti* (Aconoidasida: Piroplasmida) in Wild Rodents in Turkey. J. Med. Entomol..

[68] Akram I.N., Parveen T., Abrar A., Mehmood A.K., Iqbal F. (2019). Molecular detection of *Babesia microti* in dogs and cat blood samples collected from Punjab (Pakistan). Trop. Biomed..

[69] Moustafa M.A.M., Sasaki A., Shimozuru M., Nakao R., Sashika M., Yamazaki K., Koike S., Tanaka J., Tamatani H., Yamanaka M. (2020). Molecular detection of apicomplexan protozoa in Hokkaido brown bears (*Ursus arctos yesoensis*) and Japanese black bears (*Ursus thibetanus japonicus*). Parasitol. Res..

[70] Spada E., Proverbio D., Galluzzo P., Perego R., Bagnagatti De Giorgi G., Roggero N., Caracappa S. (2014). Frequency of piroplasms *Babesia microti* and *Cytauxzoon felis* in stray cats from northern Italy. BioMed Res. Int..

[71] Camacho A.T., Pallas E., Gestal J.J., Guitián F.J., Olmeda A.S., Telford S.R., Spielman A. (2003). *Ixodes hexagonus* is the main candidate as vector of *Theileria annae* in northwest Spain. Vet. Parasitol..

[72] Duh D., Petrovec M., Bidovec A., Avsic-Zupanc T. (2005). Cervids as Babesiae hosts, Slovenia. Emerg. Infect. Dis..

[73] Mathis A., Hilpertshauser H., Deplazes P. (2006). Piroplasms of ruminants in Switzerland and zoonotic significance of *Babesia*. Schweizer Archiv fur Tierheilkunde.

[74] Garafutdinov R.R., Galimova A.A., Sakhabutdinova A.R. (2020). The influence of quality of primers on the formation of primer dimers in PCR. Nucleosides Nucleotides Nucleic Acids.

[75] Fischhoff I.R., Bowden S.E., Keesing F., Ostfeld R.S. (2019). Correction to: Systematic review and meta-analysis of tick-borne disease risk factors in residential yards, neighborhoods, and beyond. BMC Infect. Dis..

[76] Jacob S.S., Sengupta P.P., Paramanandham K., Suresh K.P., Chamuah J.K., Rudramurthy G.R., Roy P. (2020). Bovine babesiosis: An insight into the global perspective on the disease distribution by systematic review and meta-analysis. Vet. Parasitol..

