# Supplementary figures and images for: Nucleic Acid Prevalence of Zoonotic Babesia in Humans, Animals and Questing Ticks, a Systematic Review and Meta-Analysis
Source: Trop Med Infect Dis. 2023 Feb 22;8(3):132. doi: 10.3390/tropicalmed8030132 (PMC10051474; doi:10.3390/tropicalmed8030132)

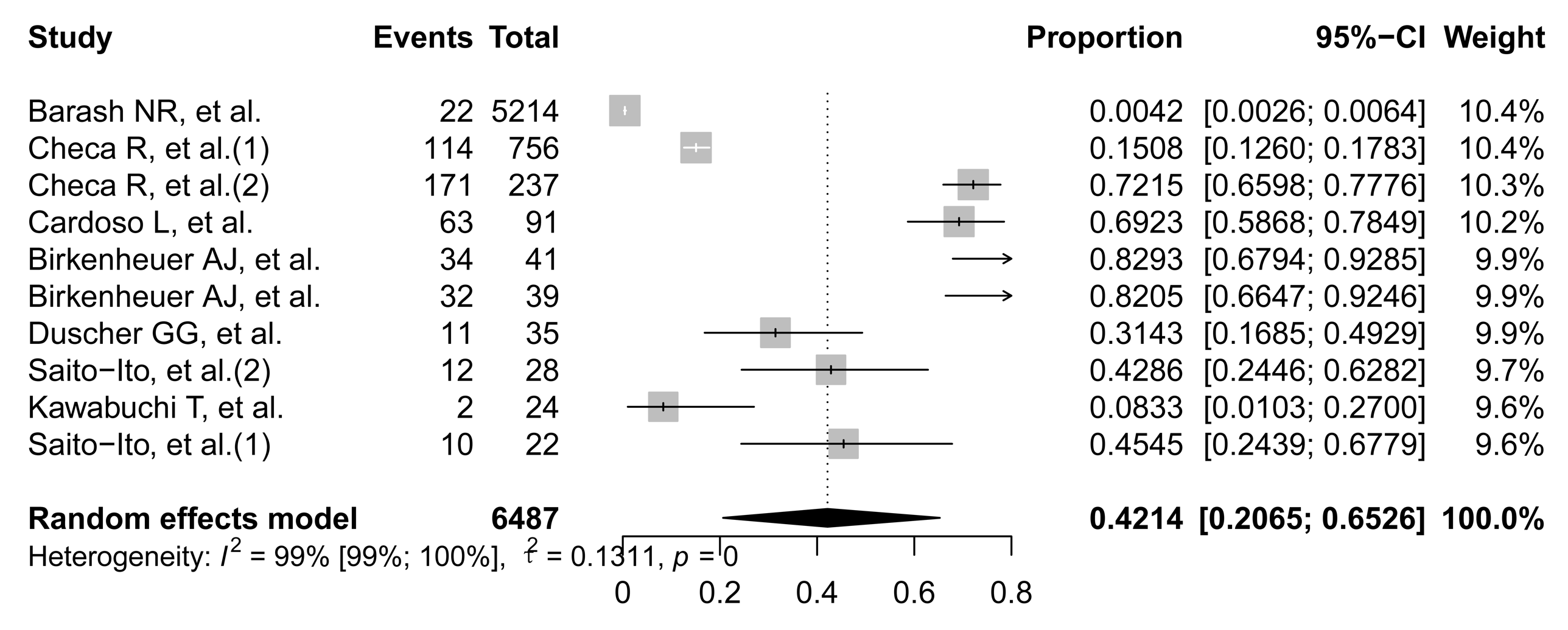

Supplement: Supplementary file 1 [file tropicalmed-08-00132-s001.zip › Nucleic acid prevalence of zoonotic Babesia in humans, animals and questing ticks, a systematic review and meta-analysis/Figure S1e. Forest plots of nucleic acid prevalence of B. microti-like in animals-01.tif]

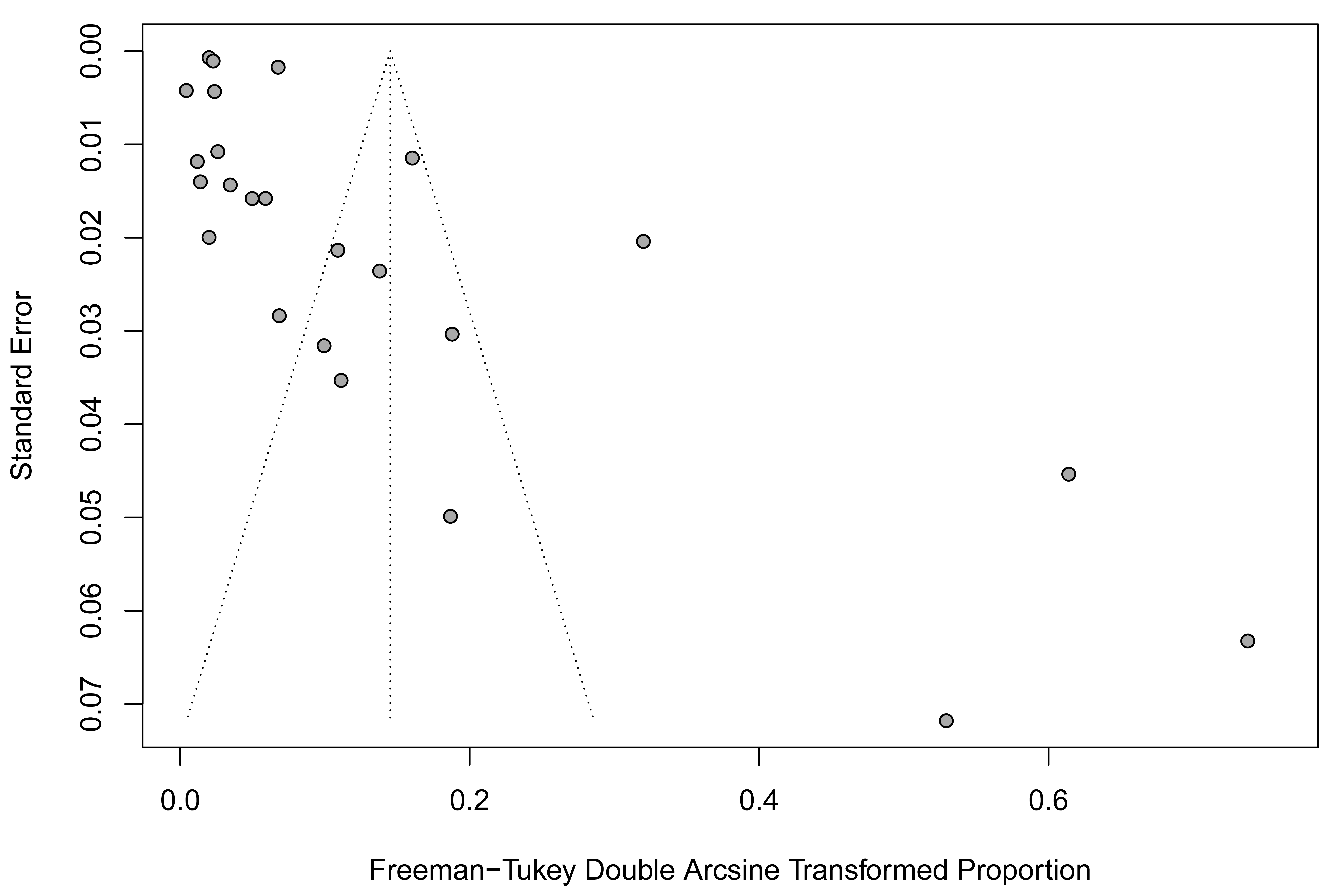

Supplement: Supplementary file 1 [file tropicalmed-08-00132-s001.zip › Nucleic acid prevalence of zoonotic Babesia in humans, animals and questing ticks, a systematic review and meta-analysis/Figure S2a. Funnel plot for assessing publication bias in studies reporting nucleic acid prevalence of B. microti in humans-01.tif]

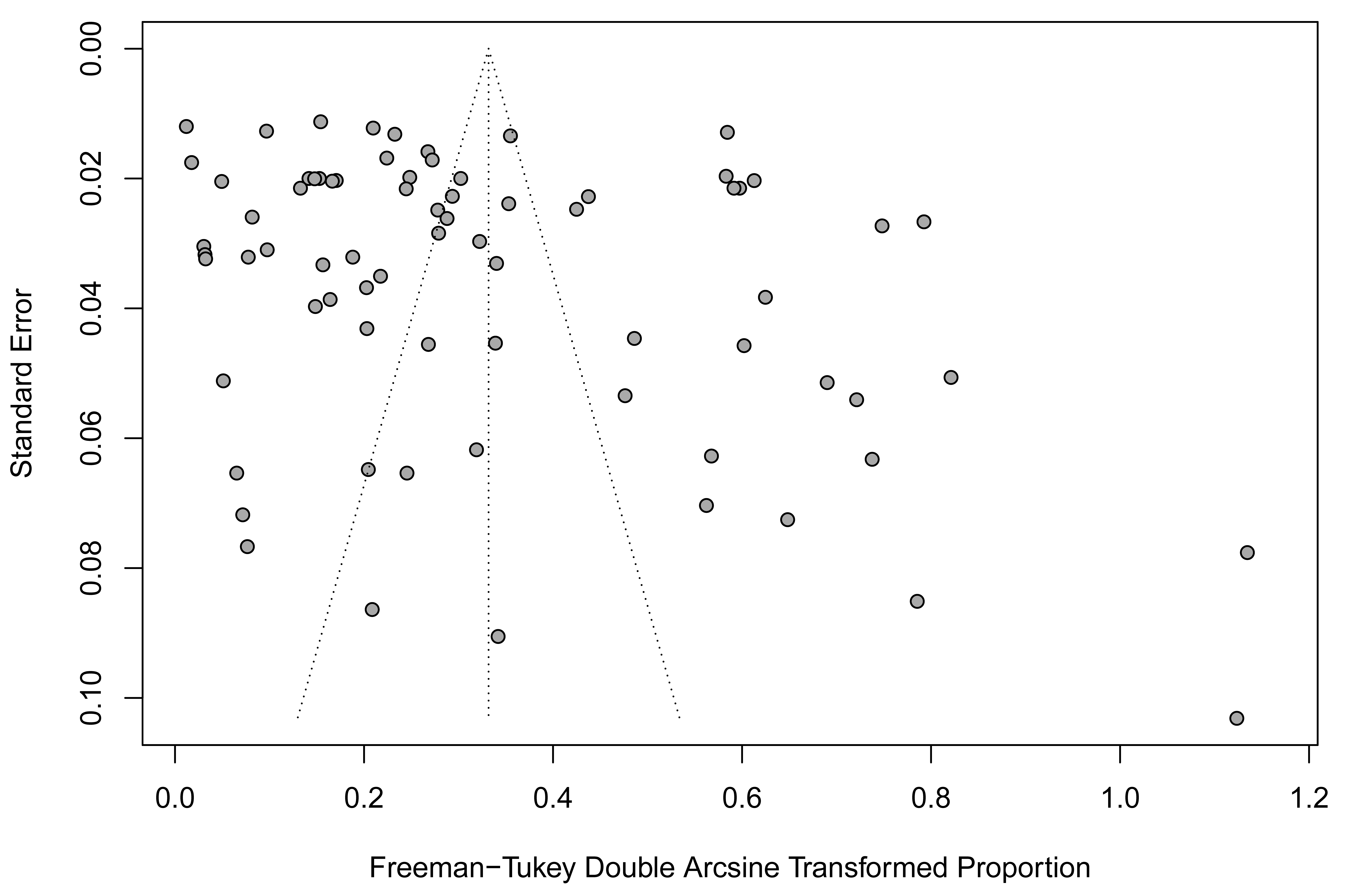

Supplement: Supplementary file 1 [file tropicalmed-08-00132-s001.zip › Nucleic acid prevalence of zoonotic Babesia in humans, animals and questing ticks, a systematic review and meta-analysis/Figure S2b. Funnel plot for assessing publication bias in studies reporting nucleic acid prevalence of B. microti in animals-01.tif]

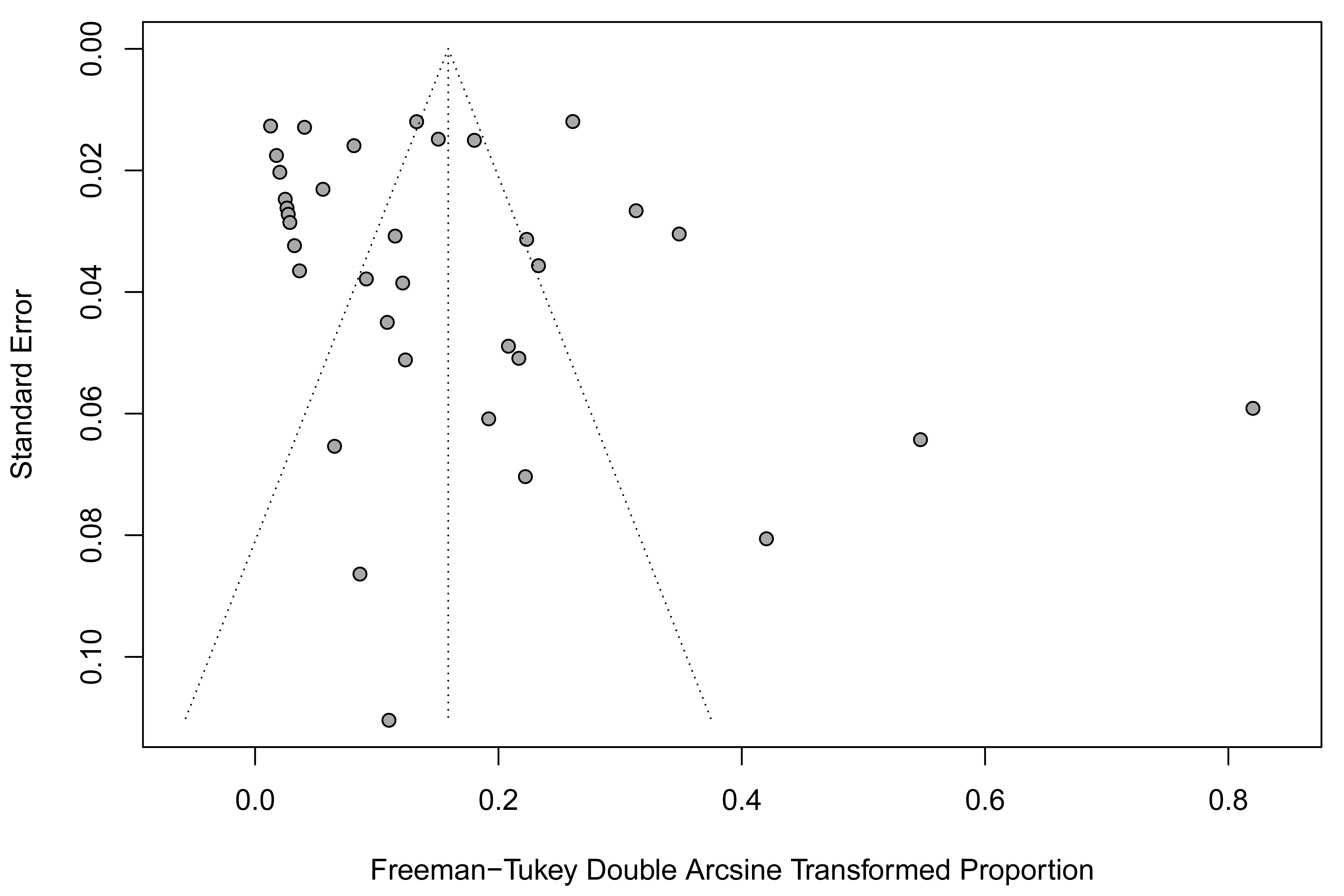

Supplement: Supplementary file 1 [file tropicalmed-08-00132-s001.zip › Nucleic acid prevalence of zoonotic Babesia in humans, animals and questing ticks, a systematic review and meta-analysis/Figure S2c. Funnel plot for assessing publication bias in studies reporting nucleic acid prevalence of B. divergens in animals-01.tif]

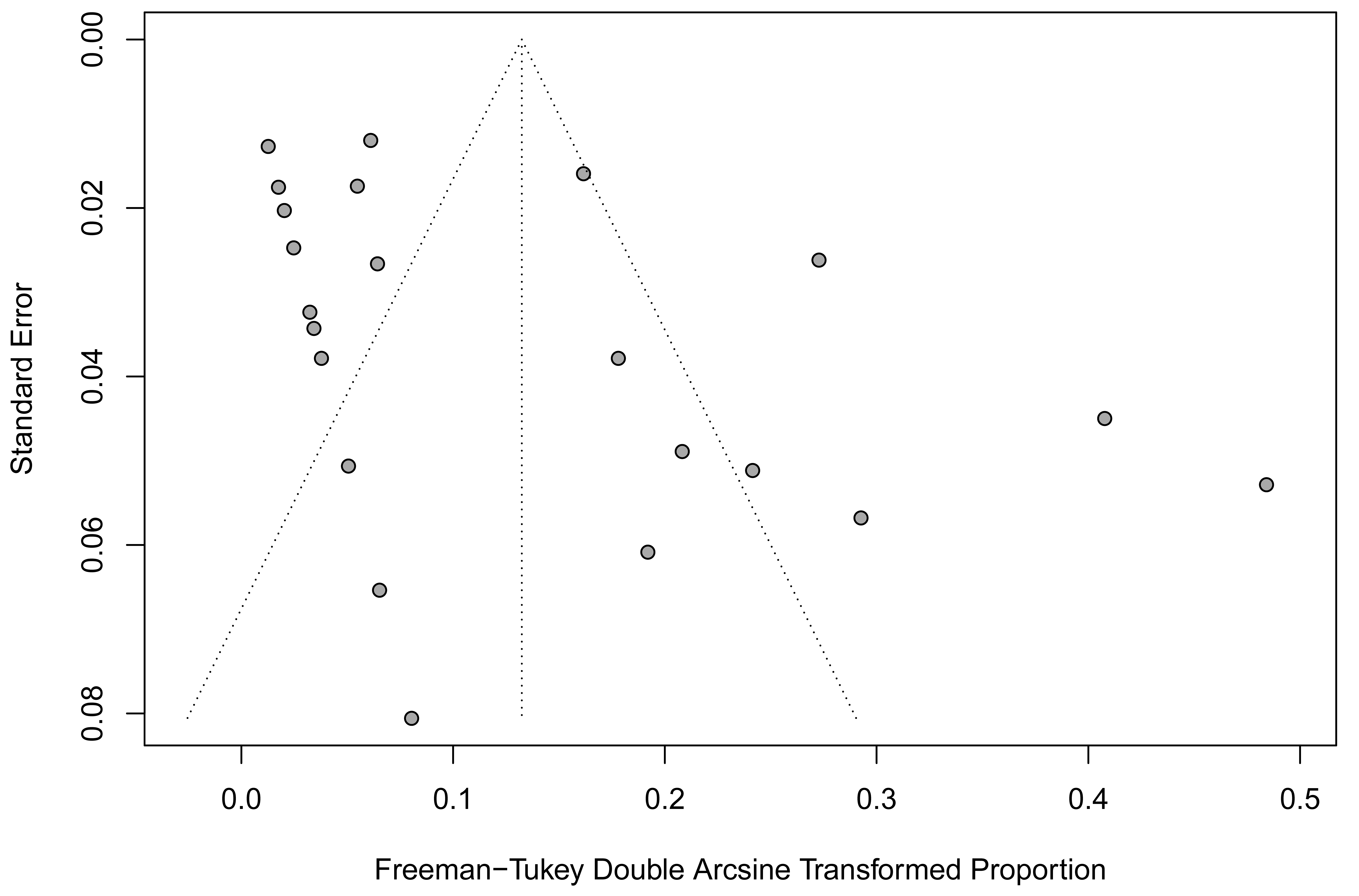

Supplement: Supplementary file 1 [file tropicalmed-08-00132-s001.zip › Nucleic acid prevalence of zoonotic Babesia in humans, animals and questing ticks, a systematic review and meta-analysis/Figure S2d. Funnel plot for assessing publication bias in studies reporting nucleic acid prevalence of B. venatorum in animals-01.tif]

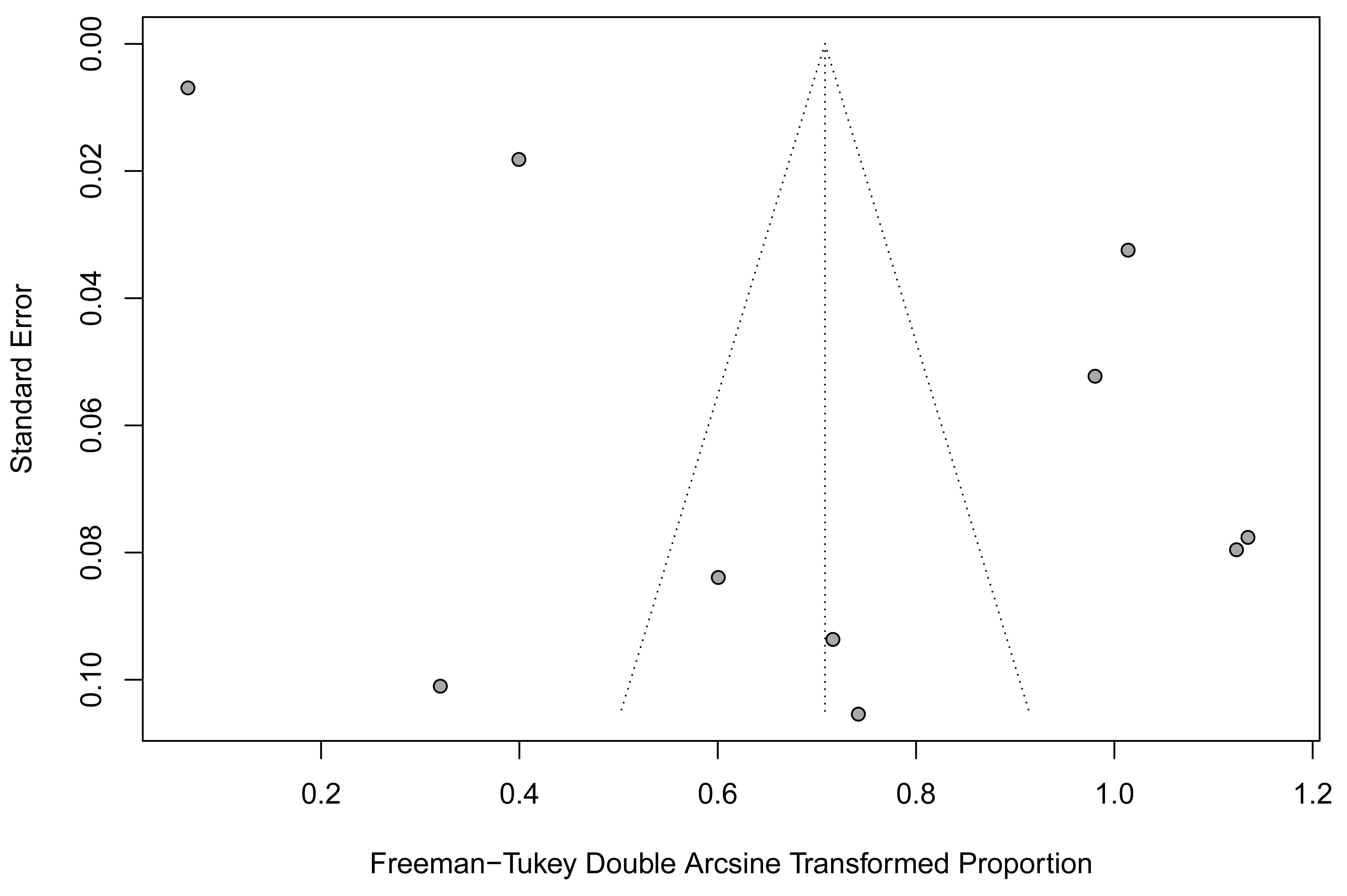

Supplement: Supplementary file 1 [file tropicalmed-08-00132-s001.zip › Nucleic acid prevalence of zoonotic Babesia in humans, animals and questing ticks, a systematic review and meta-analysis/Figure S2e. Funnel plot for assessing publication bias in studies reporting nucleic acid prevalence of B. microti-like in animals-01.tif]

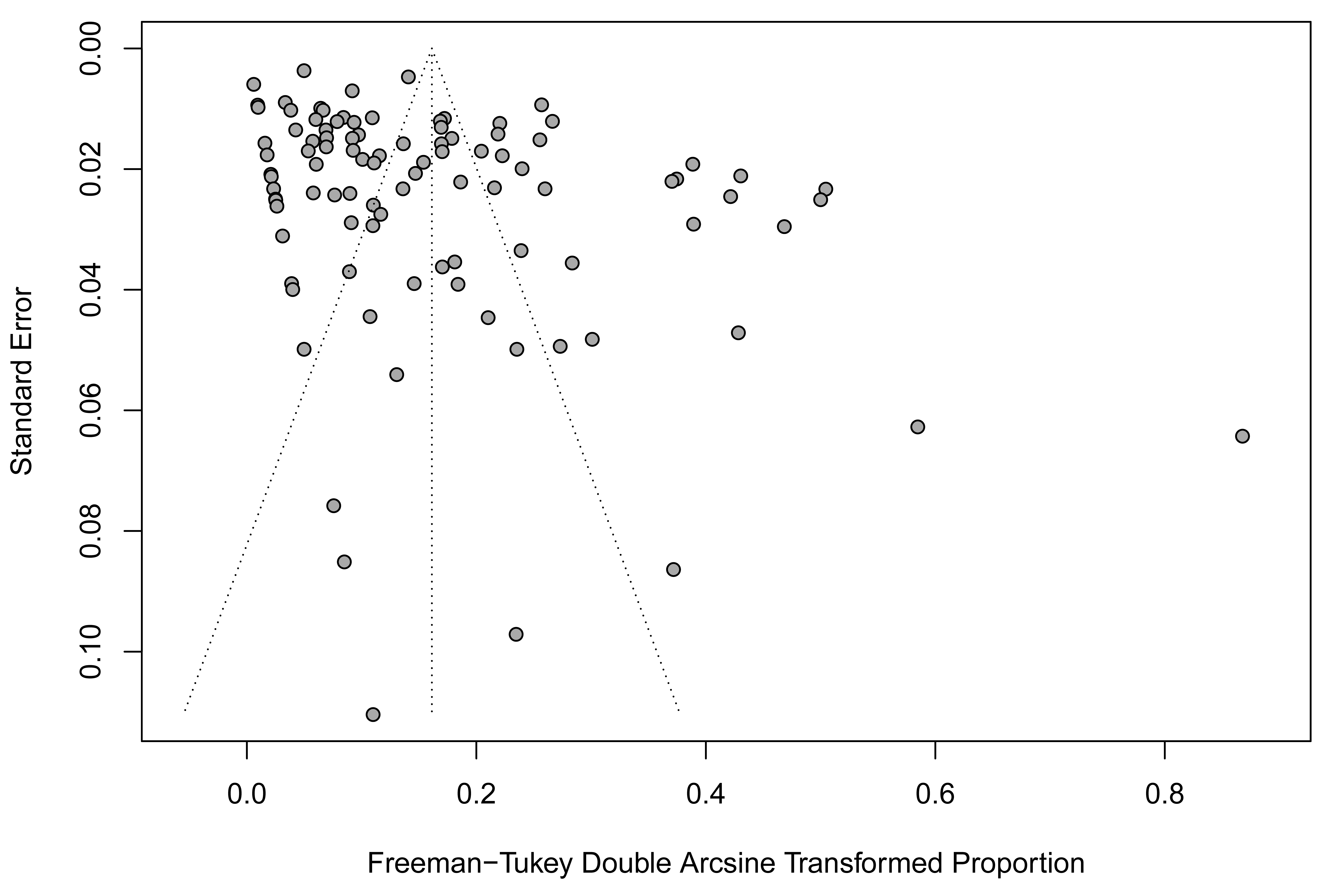

Supplement: Supplementary file 1 [file tropicalmed-08-00132-s001.zip › Nucleic acid prevalence of zoonotic Babesia in humans, animals and questing ticks, a systematic review and meta-analysis/Figure S2f. Funnel plot for assessing publication bias in studies reporting nucleic acid prevalence of B. microti in questing ticks-01.tif]

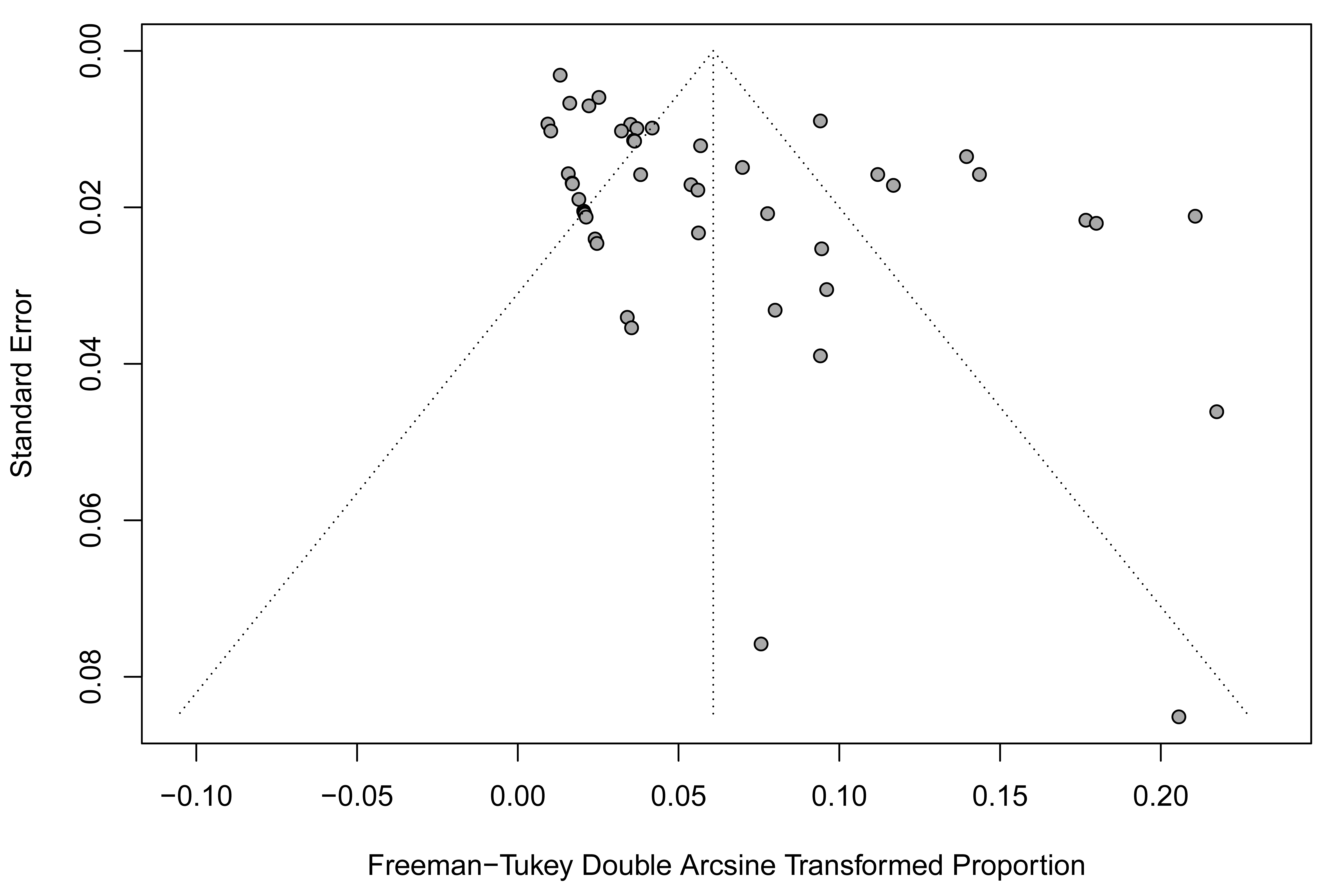

Supplement: Supplementary file 1 [file tropicalmed-08-00132-s001.zip › Nucleic acid prevalence of zoonotic Babesia in humans, animals and questing ticks, a systematic review and meta-analysis/Figure S2g. Funnel plot for assessing publication bias in studies reporting nucleic acid prevalence of B. divergens in questing ticks-01.tif]

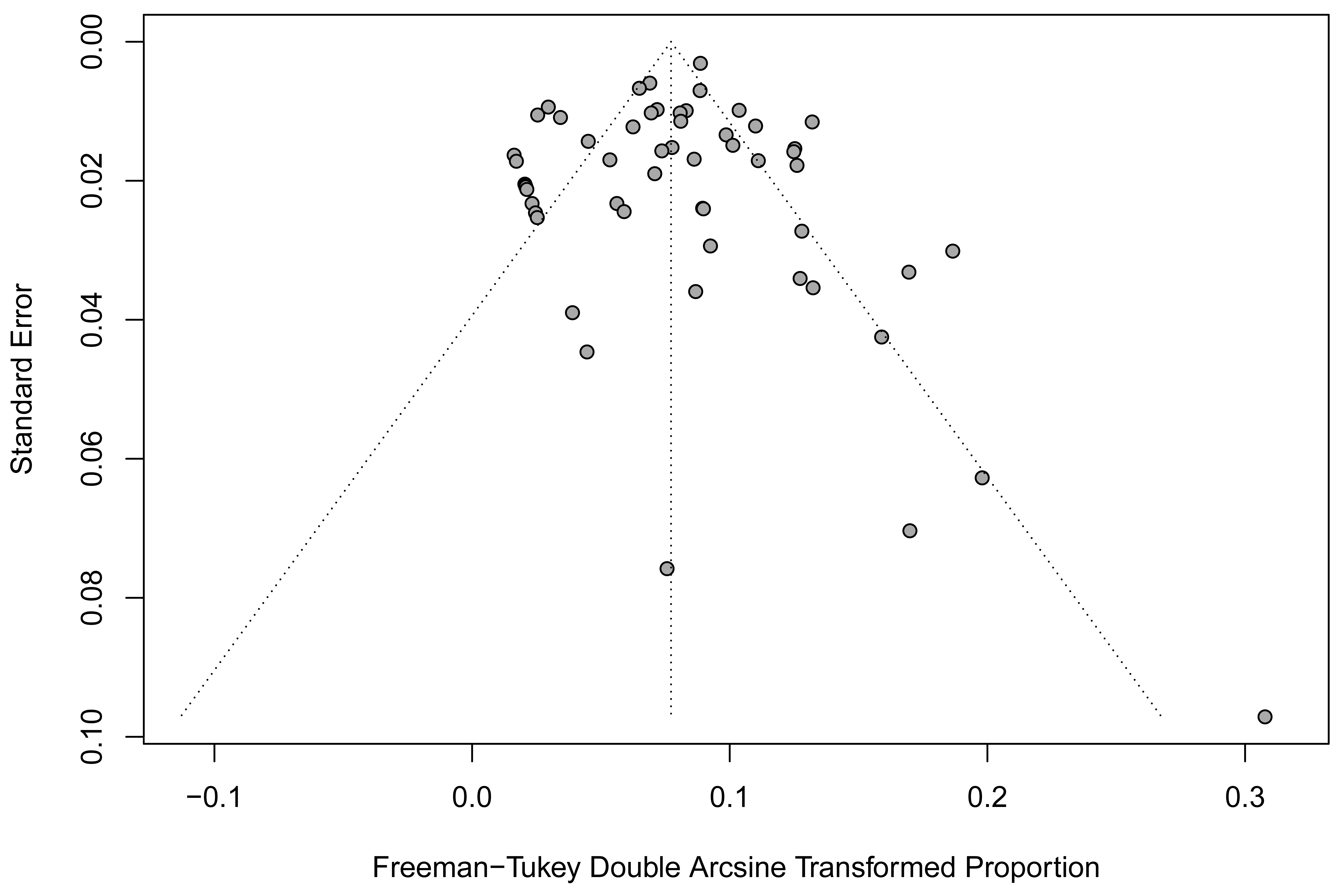

Supplement: Supplementary file 1 [file tropicalmed-08-00132-s001.zip › Nucleic acid prevalence of zoonotic Babesia in humans, animals and questing ticks, a systematic review and meta-analysis/Figure S2h. Funnel plot for assessing publication bias in studies reporting nucleic acid prevalence of B. venatorum in questing ticks-01.tif]
